# Pancreatic Stellate Cells Serve as a Brake Mechanism on Pancreatic Acinar Cell Calcium Signaling Modulated by Methionine Sulfoxide Reductase Expression

**DOI:** 10.3390/cells8020109

**Published:** 2019-02-01

**Authors:** Jin Shuai Liu, Zong Jie Cui

**Affiliations:** Institute of Cell Biology, Beijing Normal University, Beijing 100875, China; liujinshuai21@163.com

**Keywords:** pancreatic stellate cells, methionine sulfoxide reductases, co-culture inhibition, AR4-2J, calcium oscillations

## Abstract

Although methionine sulfoxide reductase (Msr) is known to modulate the activity of multiple functional proteins, the roles of Msr in pancreatic stellate cell physiology have not been reported. In the present work we investigated expression and function of Msr in freshly isolated and cultured rat pancreatic stellate cells. Msr expression was determined by RT-PCR, Western blot and immunocytochemistry. Msr over-expression was achieved by transfection with adenovirus vectors. Pancreatic stellate cells were co-cultured with pancreatic acinar cells AR4-2J in monolayer culture. Pancreatic stellate and acinar cell function was monitored by Fura-2 calcium imaging. Rat pancreatic stellate cells were found to express MsrA, B1, B2, their expressions diminished in culture. Over-expressions of MsrA, B1 or B2 were found to enhance ATP-stimulated calcium increase but decreased reactive oxygen species generation and lipopolysaccharide-elicited IL-1 production. Pancreatic stellate cell-co-culture with AR4-2J blunted cholecystokinin- and acetylcholine-stimulated calcium increases in AR4-2J, depending on acinar/stellate cell ratio, this inhibition was reversed by MsrA, B1 over-expression in stellate cells or by Met supplementation in the co-culture medium. These data suggest that Msr play important roles in pancreatic stellate cell function and the stellate cells may serve as a brake mechanism on pancreatic acinar cell calcium signaling modulated by stellate cell Msr expression.

## 1. Introduction

Reactive oxygen species (O_2_^.^, H_2_O_2_, OH^.^, ^1^O_2_, HClO) can all oxidize both structural and signaling proteins, particularly at the sulfur-containing cysteine (Cys) and methionine (Met) residues [1,2,3]. Met residue oxidation to Met sulfoxide [Met(O)] often results in major changes in protein activity: activation of calcium/calmodulin-dependent protein kinase II and of BK channels, inhibition of Kv channels, inhibition of the fibrillation process of apolipoproteins, β-amyloid peptide, α-synuclein [2] and loss of calcium sensitivity in calcium-sensing protein domains [4].

Met oxidation to Met(O) is reversed by the enzyme methionine sulfoxide reductase (Msr): MsrA reduces methionine-(S)-sulfoxide, MsrB reduces methionine-(R)-sulfoxide, back to Met [5,6]. Mammals have at least 4 separate genes (*msrA*, *msrB1-3*) [1,2,7] to encode MsrA [8,9], MsrB1 [9,10], MsrB2 [11], MsrB3 [12].

The enzyme Msr has been found to play important roles in cellular physiology and pathophysiology. MsrA may be able to alleviate multiple disorders such as bipolar disorder [13], diabetes [14], cardiac dysfunction [15], ischemia/reperfusion injury in kidney [16,17], neuroinflammation [18], atherosclerosis [19]. Cell permeant PEP-1-MsrA after entering cell interiors could readily reduce intracellular reactive oxygen species (ROS) generation, inhibit apoptosis and decrease TNFα/IL-1β mRNA contents in macrophages [19]. The protein Mical and MsrB1 together regulate actin assembly reversibly by Met oxidation/reduction [20]. Oxidized amyloid monomers are readily reduced by MsrA and B2, although Met-oxidized amyloid fibrils are poor Msr substrates [21]. ER-targeted MsrB3 affords ready protection against ER stress [12].

In chronic pancreatitis the pancreatic parenchyma is gradually replaced by fibroid tissue, leading to chronic pain and a loss of exocrine/endocrine functions [22,23]. Pancreatic fibrosis is largely due to activation or transition of pancreatic stellate cells [22,23], from the quiescent state to a myofibroblast-like phenotype, to secrete excessive extracellular matrix proteins [24]. Pancreatic stellate cells may also be critical to form the microenvironmental niche for pancreatic carcinogenesis, invasion and metastasis [25,26,27,28,29].

Although Msr is found to be expressed in a variety of cells and tissues, whether Msr is expressed and plays any role in pancreatic stellate cell function is not known. Therefore in the present work Msr expression and function in the isolated and primarily cultured rat pancreatic stellate cells were investigated. It was found that MsrA, B1, B2 were expressed in rat pancreatic stellate cells which diminished upon activation in culture. Msr over-expression reduced stellate cell inflammatory cytokine synthesis and ROS production but enhanced ATP-induced calcium responses. Pancreatic stellate cell co-culture with pancreatic acinar cell AR4-2J were found to inhibit cholecystokinin (CCK)- and acetylcholine (ACh)-induced calcium oscillations in AR4-2J cells, this inhibition was reversed by over-expression of MsrA, B1 in the co-cultured pancreatic stellate cells and by Met supplementation in the co-culture medium. Therefore Msr play an essential role in rat pancreatic stellate cell function. Importantly, Msr expression level directly modulates the inhibitory effect of pancreatic stellate cells on pancreatic acinar cell calcium signaling. In view of the strategic location of the stellate cells surrounding the basal plasma membrane of the acinar cells where all the acinar cell surface receptors are located, we suggest that pancreatic stellate cells provide a brake mechanism on pancreatic acinar cell surface receptor activation and calcium signaling. These new findings have important implications for the therapy of chronic pancreatitis and possibly also of pancreatic cancer.

## 2. Materials and Methods

### 2.1. Materials

Cholecystokinin octapeptide sulfate (CCK) was from Tocris Cookson (Bristol, UK). DMEM/F12 (1:1), trypsin (0.25% trypsin with EDTA), penicillin/streptomycin and Trizol were from Invitrogen (Shanghai, China). Acetylcholine (ACh) and bovine serum albumin (BSA) were from Sigma Aldrich (St. Louis, MO, USA). The Goods buffer 4-(2-hydroxyethyl)-1-piperazineethane-sulfonic acid (HEPES) was from Calbiochem (Darmstadt, Germany). Fura-2 AM was from AAT Bioquest (Sunnyvale, CA, USA). Nycodenze was purchased from Axis-Shield (Oslo, Norway). Cell-Tak and Agar (Bacto TM) were from BD Biosciences (Bedford, MA, USA). HRP-conjugated goat anti-rabbit IgG, HRP-conjugated goat anti-mouse IgG, monoclonal mouse anti-β-actin, PVDF membrane, the BCA protein assay kit and the ECL Western blot kit were from Beijing Kangweishiji (Beijing, China). Collagenase P, DNase I and pronase were from Roche (Beijing, China). TRITC-labeled secondary antibody (donkey anti-rabbit), DyLight 488-labeled secondary antibody (donkey anti-goat), antibodies against MsrA, MsrB1, MsrB2, MsrB3, GFAP, α-smooth muscle actin were all from Abcam (Cambridge, UK). Hoechst 33342 were from Dojindo (Shanghai, China). GoScript reverse transcription kit and GoTaq^®^ qPCR MasterMix were from Promega (Shanghai, China). pAD-kan-MsrA, pAD-kan-MsrB1, pAD-kan-MsrB2 were bought from Vigene Biosciences (Rockville, MD, USA). Rat IL-1β and IL-6 ELISA kits were from Dakewe (Beijing, China). The CellROX^®^ Oxidative Stress Reagent (Deep Red) was from Thermo Fisher Scientific (Waltham, MA, USA).

### 2.2. Isolation and Culture of Rat Pancreatic Stellate Cells, Culture of AR4-2J Cells

Rat pancreatic stellate cells were isolated by density gradient centrifugation, with Nycodenze, Iodixanol or Percoll as the density medium [30,31,32]. Briefly, the rat of the Sprague-Dawley strain (250–350 g, from HuaFuKang or Vital River Experimental Animals, Beijing, China) was killed by CO_2_ asphyxia, the pancreas was excised in a laminar flow hood, buffer (containing collagenase P 0.8 g·L^−1^, prorease 0.4 g·L^−1^, DNAase 0.3 g·L^−1^, 10 mL in volume) was infiltrated and digested for 30 min in a shaking water bath (37 °C, 120 cycles/min). Digested tissue was dispersed with a plastic pipette, filtered (150 mesh) onto buffer containing BSA 4% and centrifuged (1000× *g*, 5 min). The cell pellet was washed, before 4 mL Nycodenze (28.7%) was added, additional buffer was added to a total volume of 8.5 mL, then mixed. On top of this mixture was added 3 mL of fresh buffer, before centrifugation (1400× *g*, 4 °C, 20 min). After centrifugation, floating pancreatic stellate cells at the interface of the two layers of solutions were collected, washed, re-suspended in DMEM with 10% FBS and antibiotics and cultured at a cell density of 5 × 10^4^/cm^2^. Cell culture medium was changed 15 h later and subsequently replaced every 2 days. The pancreatic stellate cells were passaged every 3 days. This protocol was approved by The Animal Ethics Committee (CLS-EAW-2017-015) at Beijing Normal University School for Life Sciences.

The pancreatic acinar tumor cell AR4-2J was bought from ATCC (Rockville, Maryland, USA) and cultured in DMEM/F12 (1:1) (Ham’s F-12:Dulbecco’s modified Eagle’s medium at ratio of 1:1), supplemented with 20% fetal bovine serum (Gibco), antibiotics, in a CO_2_ incubator under an atmosphere of 5% CO_2_/95% air at 37 °C as reported before [33,34,35,36].

### 2.3. Measurement of Cytosolic Calcium Concentration and of ROS

Rat pancreatic stellate cells were loaded with Fura-2 AM (final concentration 10 μM) at 37 °C in a shaking water bath at 50 cycles/min for 30 min. Fura-2 AM-loaded cells were then attached to the glass cover-slips forming the bottom part of Sykes-Moore perfusion chambers. The cover-slips were coated previously with Cell-Tak (0.6 g·L^−1^, 1 μL to each cover-slip). For cultured AR4-2J cells or AR4-2J cells co-cultured with pancreatic stellate cells, attached cells together with cover-slips were assembled into the Sykes-Moore perfusion chambers, Fura-2 AM at a final concentration of 10 μM were added and loaded for 30 min minimum before perfusion and experimentation.

Calcium concentrations were measured as reported previously in a Photon Technology International (PTI, now HORIBA Scientific, Edison, New Jersey, NJ, USA) calcium measurement system [35,36,37,38,39]. The PTI calcium measurement system was hooked to an inverted fluorescent microscope Olympus IX 70 or Nikon TE-2000U, with a PMT (PTI PMT814) or CCD (NEO-5.5-CL-3, Andor) as detector. Fura-2 was excited alternately at 340 nm/380 nm by light from monochromater DeltaRam V or X. Fluorescence intensity or images were detected/collected after filtering at 510 ± 25 nm. Fluorescence ratios F_340_/F_380_ were plotted against time by SigmaPlot as indicative of cytosolic calcium concentration changes [35,36,37,38,39,40,41].

Pancreatic stellate intracellular ROS contents were measured in a flow cytometer (NovoCyte^®^3000, ACEA Bioscience, San Diego, CA, USA). Briefly, cells (5 × 10^4^) were planted in 6-well plates, allowed to attach overnight, before staining with CellROX Deep Red 5 µM at 37 °C for 30 min. CellROX-loaded cells were then detached with trypsin digestion, collected, re-suspended in a flow cytometer tube (5 mL round bottom tubes, Corning, NY, USA), fluorescence intensity was analyzed in NovoCyte^®^3000, with λ_ex_ 640 nm, λ_em_ 665 nm. Data were plotted by software NovoExpress^®^ as curves of cell number against fluorescence intensity.

### 2.4. Reverse Transcription-PCR and Real Time Quantitative PCR (RT-qPCR)

Total mRNA from pancreatic stellate cells was extracted with Trizol as described in manufacturer’s instructions. cDNA was generated using GoScript reverse transcription kit (Promega). Single-strand cDNA was synthesized from 2 μg RNA. The first-strand product (2 μL) was used as template in each PCR reaction (in a volume of 20 μL), under the following conditions: initial de-naturation at 94 °C for 5 min, PCR cycles: 94 °C for 30 s, 57 °C for 30 s, 72 °C for 1 min repeated for 32 cycles, final elongation at 72 °C for 5 min. PCR products were resolved on 2% agarose gel and analyzed by Gel Imager. Primers: MsrA-forward: CCGTAGCAGCCAAACA, MsrA-reverse: TGGGTCGGGTCGTGAT; MsrB1-forward: AGACCTGAGGGCTTTACTTG, MsrB1-reverse: CATTGAGGAACTCGTGGC; MsrB2-forward: TAACTCACTTGGCAGCAC, MsrB2-reverse: CGGCACGACTCATAGC; GAPDH-forward: GTGGAGTCTACTGGCGTCTT, GAPDH-reverse: CCAGGATGCCCTTTAGTG.

For real-time quantitative PCR (RT-qPCR), mRNA was extracted with Trizol as described in manufacturer’s instructions. cDNA was generated using GoScript reverse transcription kit (Promega). Expression of mRNA was determined using GoTaq^®^ qPCR MasterMix. Reactions were run on an ABI 7500 Real-Time PCR machine (Applied Biosystems) in triplicates (in a volume of 20 μL) using standard machine settings. Expression data were normalized using β-actin mRNA as endogenous reference and relative expression values were calculated. Primers: IL-1-forward: GAC CCCAAAAGATTAAGGATT, IL-1-reverse: AAAGAAGGTGCTTGGGTCCTC; Jak-forward: TTTGAAGACAGGGACCCTACACAG, Jak-reverse: TCATAGCGGCACATCTCCACA; STAT3-forward: TTTGAGACAGAGGTGTACCACCAAG, STAT3-reverse: ACCACAGGATTGATGCCCAAG; β-actin-forward: CCCATCTATGAGGGTTACGC, β-actin-reverse: TTTAATGTCACGCACGATTTC.

### 2.5. Over-Expression of Msr in Cultured Pancreatic Stellate Cells

The pAD-kan-MsrA, pAD-kan-MsrB1, pAD-kan-MsrB2 from Vigene Biosciences (Rockville, MD, USA) were digested (37 °C, 3 h) with PacI enzyme (restriction digest buffer 5 µL, ORF in pEnter 2 µg/20 µL, PacI 1 µL, nuclease-free water 24 µL), before enzyme inactivation (80 °C, 15 min). HEK293T cells (3–5 × 10^6^) were planted in T75 culture flask one day before transfection with 2 µg linerized pAD-kan-Msr DNA. Four to five days later, the cell monolayer was examined for cytopathic effects. When cytopathic effect is nearly complete (i.e., most cells rounded up but not yet detached), cells were detached by pipetting and harvested. Harvested cells were pooled before centrifugation (1000× *g* for 5 min) with the cell pellet re-suspended in medium or in Tris buffer (Tris 10 mM, NaCl 100 mM, pH 8.0, 0.25–0.5 mL per T75 flask). Adenoviruses were released in 3 freeze/thaw cycles, before centrifugation (3000× *g* for 10 min); the supernatant viral stock was stored at –80 °C or immediately purified and tittered for use.

Pancreatic stellate cells were grown in six-well plates to 70% confluence before adenoviral stock (10^12^ vg·mL^−1^ × 5 uL) was added; cells were then cultured and used for experiments on desired days.

### 2.6. ELISA and Western Blot

IL-1β protein content in rat pancreatic stellate cell supernatant/medium was quantified by an ELISA kit according to the manufacturer’s instructions.

For Western blot, rat pancreatic stellate cells were lysed in ice-cold lysis buffer (Tris 50 mM, NaCl 150 mM, NP-40 1%, SDS 0.1%, PMSF 1 mM, pH 7.4). Total protein was quantified using BCA protein quantification kit. Equal amounts of protein (lysate) were loaded in each lane and separated on 10–15% SDS/PAGE gels. Proteins (20 μg in each lane) were separated electrophoretically before transfer onto PVDF membranes. After blocking with 5% milk, membranes were incubated with primary antibodies overnight at 4 °C. Appropriate peroxidase-conjugated secondary antibodies were added and peroxidase reaction proceeded. Blots were developed and protein contents were quantified by enhanced chemiluminescence (ECL).

### 2.7. Immunocytochemistry

Pancreatic stellate cells grown on cover-slips were washed in phosphate buffered saline, fixed in 4% paraformaldehyde (10 min), permeabilized in 0.2% Triton X-100 (15 min), blocked in 3% BSA (60 min). Fixed cells were then incubated with primary antibodies against MsrA, B1, B2 or α-smooth muscle actin (SMA) at 4 °C overnight, washed, incubated with secondary antibodies at room temperature for 30 min before wash. The cells were then counterstained with Hoechst 33342 for 15 min and washed. The wash after incubation with primary and secondary antibodies and with Hoechst was done in phosphate buffered saline containing Triton X-100 0.2%, Tween-20 2%. The slide was then sealed and imaged in a confocal microscope (Zeiss LSM 510 META) under an objective of 63×/1.40 oil. The secondary antibodies were TRITC-labeled donkey anti-rabbit or DyLight 488-labeled donkey anti-goat secondary antibodies, with TRITC λ_ex_ 543 nm, λ_em_ 572 nm, DyLight 488 λ_ex_ 488 nm, λ_em_ 518 nm, respectively.

### 2.8. Data Analysis and Statistics

All experiments were done at least 3 times as indicated. Data were presented in mean ± SEM and plotted with SigmaPlot. Data analysis was done with Student’s *t* test, statistical significance at *p* < 0.05 was indicated with an asterisk (*).

## 3. Results

### 3.1. Msr Expression in Rat Pancreatic Stellate Cells

**Figure 1:**
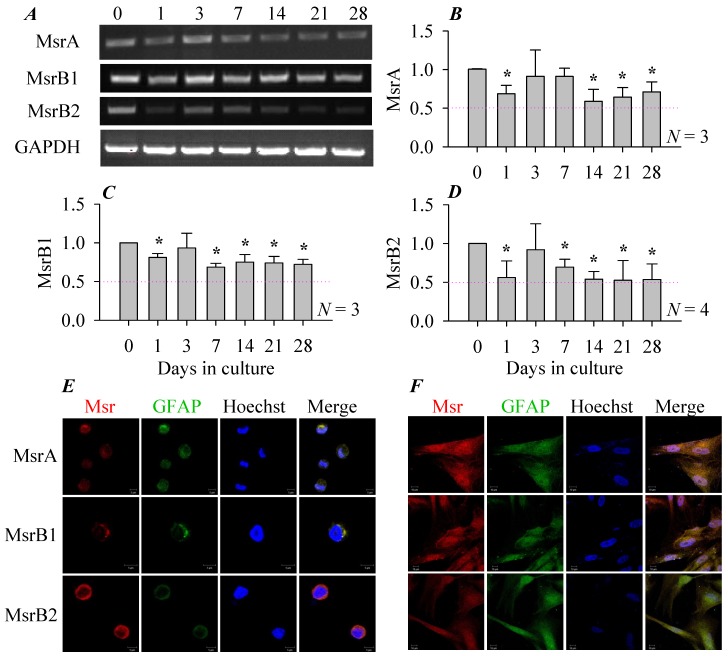
Msr expression in rat pancreatic stellate cells. RT-PCR was done as described in *Methods* and representative original images (**A**) and averaged Msr mRNA contents (**B**–**D**) are shown for rat pancreatic stellate cells cultured for 0, 1, 3, 7, 14, 21, 28 days. All data are expressed as Msr/GAPDH ratios normalized to that on day of isolation (day 0) (mean ± SEM, *N* = 3). The thin pink dashed lines indicate ratio 0.5. Asterisks (*) indicate statistical significance at *p* < 0.05, *N* ≥ 3 as indicated. The freshly isolated (**E**) or primary cultured (for 14 days) (**F**) rat pancreatic stellate cells were fixed and immunocytochemistry done. Primary antibody: anti-MsrA, B1, B2, GFAP; secondary antibody: TRITC-tagged donkey anti-rabbit, DyLight 488-tagged donkey anti-goat antibody. Nucleus was counter-stained with Hoechst 33342. Confocal images were taken in a Zeiss LSM 510 META (objective 63×/1.40 oil), with λ_ex_ TRITC/543 nm, Hoechst 33342/405 nm. No fluorescence was detected in controls (without primary and secondary antibodies, without primary antibody but with secondary antibody, non-specific rabbit IgG as primary antibody and secondary antibody, not shown). The band location for MsrA, B1, B2 and GAPDH is 301, 308, 234, 537 bp respectively in (**A**). The scale bars are 5 μm in (**E**) and 10 μm in (**F**).

RT-PCR measurements of mRNA contents of Msr revealed that MsrA, B1, B2 were all expressed in the freshly isolated rat pancreatic stellate cells, the expression level gradually decreased with time in culture, which was up to 4 weeks after isolation (Figure 1A). Msr expression probably recovered on day 3 in culture and also on day 7 in the case of MsrA, otherwise MarA, B1, B2 all decreased at 1, (7), 14, 21 and 28 days in culture (Figure 1B–D). MsrB2 mRNA expression in particular fell to about only half the initial values (at isolation) after culture of 2–4 weeks (Figure 1D). The second phase of decreased Msr expression on day 7 followed the expression of α-smooth muscle actin (i.e., pancreatic stellate cell activation) which occurred on day 5 (data not shown).

Immunocytochemistry confirmed that MsrA, B1, B2 were all expressed both in the freshly isolated (Figure 1E) and in cultured (14 days, Figure 1F) rat pancreatic stellate cells. Both phenotypes were found to express MsrA, B1, B2 (Figure 1E,F) but not MsrB3 (not shown). MsrA, B1, B2 were present mainly in the cytoplasm in the freshly isolated rat pancreatic stellate cells (Figure 1E). In culture, MsrA, B1, B2 were found in the cytoplasm but also in the nucleus (Figure 1F). Since Msr content all seemed to decrease after activation in culture, to elaborate their specific functions, we over-expressed MsrA, B1, B2 in the cultured pancreatic stellate cells with adenovirus vectors.

### 3.2. Msr Over-Expression in Rat Pancreatic Stellate Cells

**Figure 2:**
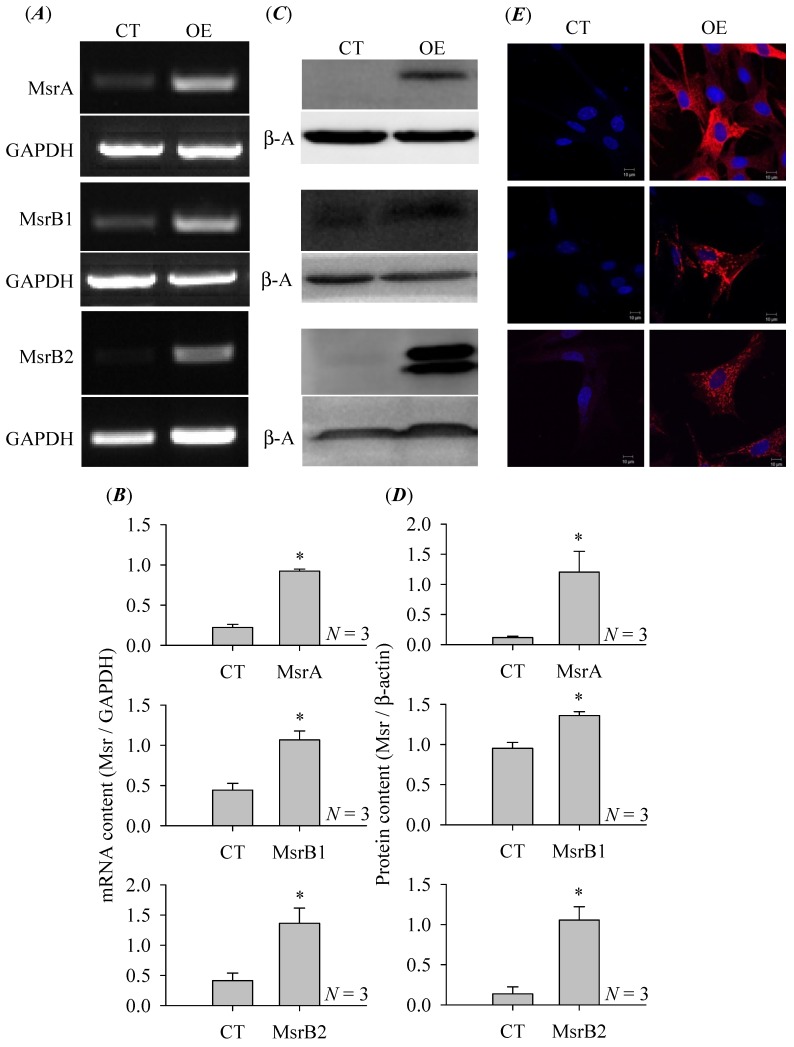
Msr over-expression in cultured rat pancreatic stellate cells. Pancreatic stellate cells were transfected with adenovirus vectors. MsrA, B1, B2 over-expression was detected by RT-PCR (**A**,**B**), Western blot (**C**,**D**) and immunocytochemistry (**E**), each both in control (CT) and over-expressing cells (OE). The primary antibodies: anti-MsrA, B1, 2, secondary antibody (TRITC labeled donkey anti-rabbit). Nucleus was counter-stained with Hoechst 33342. Imaging was done in a Zeiss LSM 510 META (objective 63×/1.40 oil) with λ_ex_: TRITC/543 nm, Hoechst 33342/405 nm. No fluorescence was observed in time-matched control experiments (without primary and secondary antibodies, without primary antibody but with secondary antibody, non-specific rabbit IgG as primary antibody plus secondary antibody, not shown). Msr expression at the mRNA (**A**) or at the protein (**C**) levels were analyzed with ImageJ and presented as ratios of Msr/GAPDH or Msr/β-actin (β-A) respectively in (**B**) and (**D**). An asterisk (*) indicates statistical significance at *P* < 0.05, with *N* = 3. Note that for optimal imaging of MsrA, B1, B2 in Msr-over-expressing pancreatic stellate cells (OE) in panel (**E**), the laser power was turned further down for time-matched parallel imaging in both control (CT) and over-expressing (OE) cells. For better images in control pancreatic stellate cells, please refer to Figure 1E,F. To see both control and overexpressed Msr immunocytochemistry simultaneously, please consult Figure A1 in Appendix A, which showed a rather overexposed staining pattern in Msr-overexpressing rat pancreatic stellate cells. The band location for MsrA, B1, B2 and GAPDH in (**A**) is 301, 308, 234, 537 bp respectively. The band location for MsrA, B1, B2 and β-actin (β-A) in (**C**) is 27, 13, 20, 42 kD respectively. The scale bars are 10 μm in (**E**).

At 24 h after infection with MsrA, B1 or B2 adenovirus vectors, RT-PCR revealed markedly enhanced mRNA contents of MsrA, B1, B2 in transfected cells than in controls (Figure 2A,B). Western blot confirmed that 24 h after infection, MsrA, B1, B2 protein contents were also significantly elevated than in control pancreatic stellate cells transfected with only GFP-containing vectors (Figure 2C,D). Immunocytochemistry done 24 h after infection similarly revealed markedly increased MsrA, B1, B2 protein contents, note the cytoplasmic puncta pattern especially for MsrB1, 2 (Figure 2E). Msr over-expression has been found to have significant effects on pancreatic stellate cell physiology as shown below.

### 3.3. Effects of Msr Over-Expression on Rat Pancreatic Stellate Cell Function

**Figure 3:**
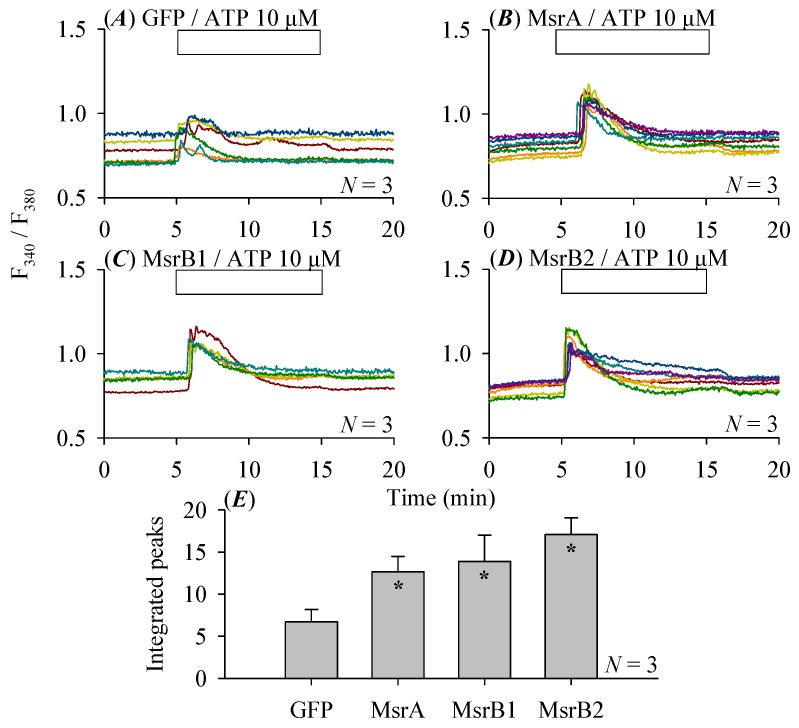

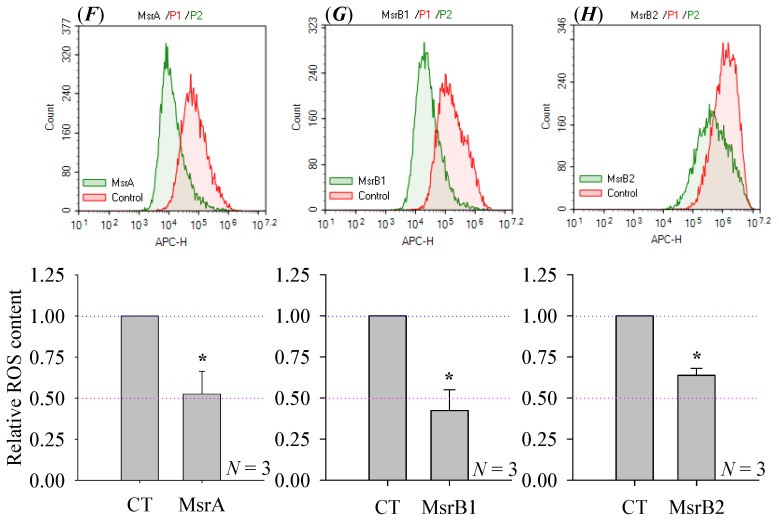
Msr over-expression enhanced ATP-stimulated calcium increases but suppressed reactive oxygen species production in pancreatic stellate cells. At 24 h after adenovirus transfection of GFP (**A**) or MsrA (**B**), MsrB1 (**C**), MsrB2 (**D**), rat pancreatic stellate cells were detached, loaded with Fura-2 AM, attached to Sykes-Moore perfusion chamber on Cell-Tak-coated glass bottom and perfused with ATP 10 μM as indicated by the horizontal bars. ATP-induced calcium responses were quantified by integrating the calcium peaks (from 5–15 min, above the basal levels) and plotted (**E**). Comparisons were made between control (GFP, **A**) and MsrA (**B**), MsrB1 (**C**), MsrB2 (**D**) over-expressing pancreatic stellate cells. Asterisks (*) indicate statistical significance at *p* < 0.05 (*N* = 3). Color-coded calcium tracings in (**A**–**D**) are each from individual pancreatic stellate cells from a representative experiment. At 36 h after adenovirus-mediated over-expression of MsrA (**F**), MsrB1 (**G**) or MsrB2 (**H**), rat pancreatic stellate cells (14 days in culture in total) were incubated with CellROX (5 μM) for 30 min (37 °C), fluorescence was detected by flow cytometry (typical charts shown in upper panels) and plotted (lower panels) (**F**–**H**). Asterisks (*) indicate statistical significance at *p* < 0.05, *N* = 3. Note the thin blue and pink horizontal dashed lines (lower panels in **F**–**H**) which indicate the levels of 1.00 and 0.50 on the ordinate respectively.

The purinergic agonist ATP induced conspicuous increases in cytosolic calcium concentration in cultured control rat pancreatic stellate cells (Figure 3A). After Msr (MsrA, B1, B2) over-expression, ATP 10 μM induced calcium responses were markedly enhanced (Figure 3B–D), at statistically significant levels (*p* < 0.05, Figure 3E).

Cultured rat pancreatic stellate cells produced a baseline level of reactive oxygen species as revealed with the fluorescent redox indicator CellROX (Figure 3F–H). Flow cytometry showed that in pancreatic stellate cells over-expressing MsrA, B1, B2 (14 days in culture in total), the content of reactive oxygen species was significantly reduced (Figure 3F–H, upper panels), down to 53%, 42%, 63% of controls (Figure 3F–H, lower panels). Other than changes in acute responses in cytosolic calcium and reactive oxygen species production, further experiments found that Msr over-expression also affected longer term responses in pancreatic stellate cells, as shown below.

**Figure 4:**
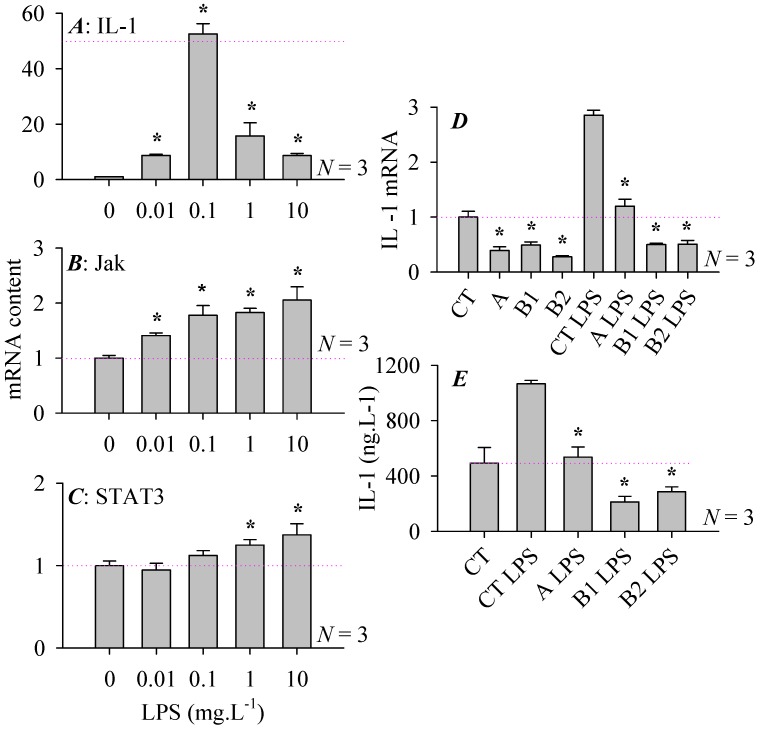
Msr over-expression inhibited LPS-stimulated IL-1 production. Cultured rat pancreatic stellate cells were stimulated with LPS (0, 0.01, 0.1, 1, 10 mg·L^−1^) for 24 h. IL-1, JAK, STAT3 mRNA content was detected by real-time quantitative PCR (**A**–**C**). At 24 h after adenovirus-mediated over-expression of MsrA, B1 or B2, rat pancreatic stellate cells were stimulated with LPS (1 mg·L^−1^) for 24 h, IL-1 expression was then measured by real-time PCR for mRNA content (**D**) or by ELISA for protein content in medium (**E**). CT: control cells with empty vector. A, B1, B2: MsrA, MsrB1, MsrB2 over-expressing cells respectively. CT: empty vector cells. CT LPS: empty vector cells stimulated with LPS. A LPS, B1 LPS, B2 LPS: MsrA, MsrB1, MsrB2 over-expressing cells stimulated with LPS respectively, all at 1 mg·L^−1^. The pink thin dashed horizontal line in (**A**) indicates mRNA level of 50, in (**B**–**D**) the mRNA level of 1.00 and in (**E**) IL-1 content from control cells (CT, empty vector). Asterisks (*) indicate statistical significance when compared with LPS concentration of 0 in (**A**–**C**), when compared with CT or CT LPS respectively in (**D**), when compared with CT LPS in (**E**), all at *p* < 0.05, *N* = 3.

Rat pancreatic stellate cells typically express the Toll-like receptor 4, which is activated by TLR4 agonist lipopolysaccharide (LPS) [42,43,44,45]. LPS (0.01, 0.1, 1, 10 mg·L^−1^) stimulation for 24 h was found to increase concentration-dependently the mRNA content of IL-1, Jak, STAT3. IL-1 mRNA production was the most responsive, with minimum effective LPS concentration of 0.01 mg·L^−1^. Maximal effect was achieved at 0.1 mg·L^−1^ but higher LPS concentrations (1, 10 mg·L^−1^) led to supra-maximal inhibition, resulting in a bell-shaped dose response curve (Figure 4A). LPS (0.01, 0.1, 1, 10 mg·L^−1^, 24 h) dose dependently stimulated Jak mRNA expression but in this case with a monophasic dose response curve (Figure 4B), also note the different scales of response (Figure 4A,B). The generation of STAT3 mRNA was the least responsive; LPS stimulated STAT3 mRNA expression only at concentrations of ≥1 mg·L^−1^ (24 h) and the increase was rather moderate (Figure 4C). Therefore IL-1 mRNA expression was the most responsive to LPS stimulation; LPS at 0.1 mg·L^−1^ elicited an increase of more than 50-fold of the baseline IL-1 mRNA content (Figure 4A).

Msr over-expression was found to have marked effect on LPS-stimulated IL-1 expression, both at the mRNA and protein levels. MsrA, B1 or B2 over-expression suppressed significantly both basal and LPS-stimulated IL-1 mRNA content (Figure 4D). ELISA assay confirmed that Msr over-expression of Msr A, B1, B2 all inhibited LPS-stimulated secretion of IL-1 into the medium (Figure 4E). Therefore Msr over-expression exerts marked effect on pancreatic stellate cell physiology. Not only that, in further experiments it was found that Msr over-expression also modulated pancreatic stellate cell/acinar cell interaction, as shown below.

### 3.4. Pancreatic Stellate Cell Co-Culture Inhibited AR4-2J Cell Calcium Signaling

**Figure 5:**
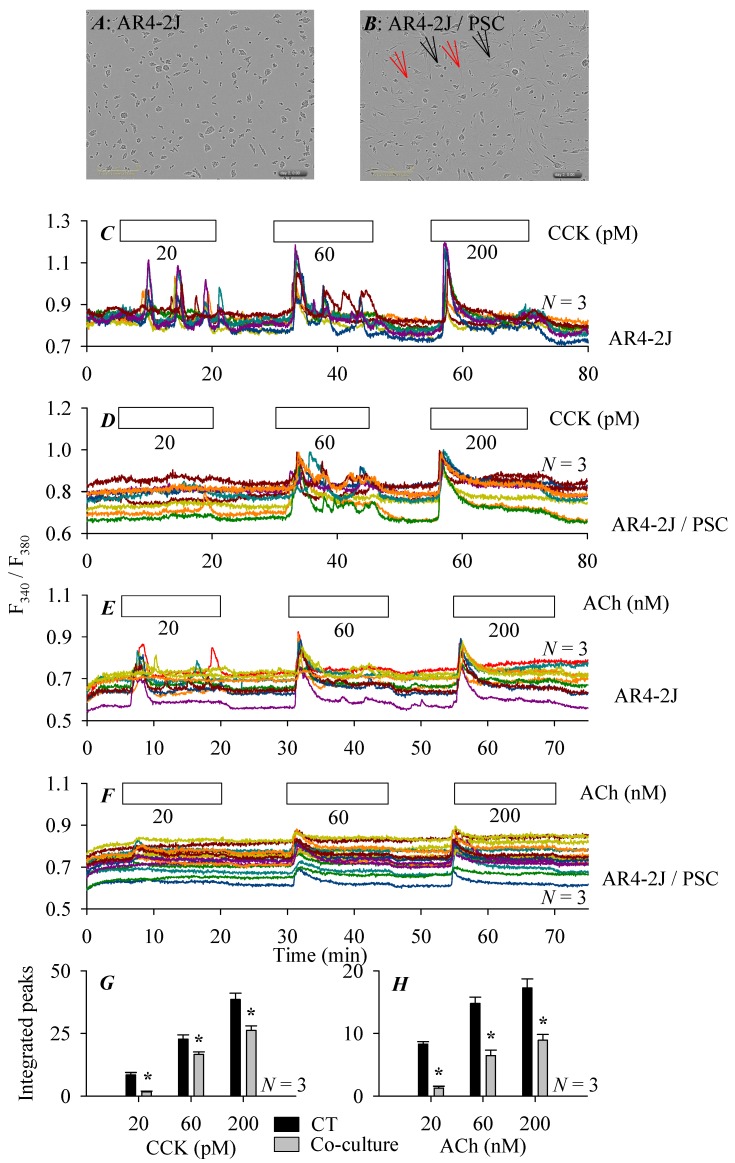
AR4-2J/PSC co-culture inhibition of CCK- and ACh-stimulated calcium increases in AR4-2J cells. AR4-2J cells cultured alone (**A**) or co-cultured with pancreatic stellate cells (at AR4-2J/PSC ratio of 2:1) (**B**) at 48 h of culture or co-culture (imaged by Incucyte). Fura-2-loaded AR4-2J (**C**,**E**) or AR4-2J/PSC (**D**,**F**) were perfused, CCK (20, 60, 200 pM, **C**,**D**), ACh (20, 60, 200 nM) (**E**,**F**) were added as indicated by the horizontal bars. Color-coded calcium tracings in each panel (**C**–**F**) were from individual AR4-2J cells in a typical experiment. The integrated calcium peaks (area under the peaks above the basal calcium levels) were quantified, CCK- (**G**) or ACh- (**H**) induced calcium responses in control (**C**,**E**) or co-cultured (**D**,**F**) AR4-2J cells were compared. Asterisk (*) indicate statistical significance at *p* < 0.05, *N* = 3. The scale bars are 300 μm in (**A**,**B**). Black and red arrows indicate AR4-2J and pancreatic stellate cells respectively in the co-culture (**B**). Enlarged versions of the micrographs (**A**,**B**) are presented as Figure A2 in Appendix A.

The rat pancreatic acinar tumor cells AR4-2J grow in clusters in culture (Figure 5A). After addition of isolated rat pancreatic stellate cells for co-culture or mixed culture (at an AR4-2J/pancreatic stellate cell ratio of 2:1), AR4-2J cells became rather flat and more spindle-shaped (Figure 5B).

AR4-2J cells express the cholecystokinin 1 receptor (CCK1R) which is coupled to the calcium signaling pathway. CCK dose-dependently (20, 60, 200 pM) stimulated calcium increases in AR4-2J cells cultured alone: CCK 20 pM induced regular calcium oscillations over the baseline, CCK 60 pM also induced calcium oscillations but with an enlarged initial spike, CCK 200 pM induced an elevated calcium plateau following a major calcium peak (Figure 5C). After co-culture with pancreatic stellate cells, CCK stimulation was markedly blunted: CCK 20 pM no longer induced multiple conspicuous calcium spikes, CCK 60 and 200 pM still triggered calcium increases, although at diminished levels (Figure 5D). ACh (20, 60, 200 nM) similarly stimulated graded calcium increases in AR4-2J cells cultured alone (Figure 5E). After co-culture with pancreatic stellate cells, ACh 20 nM almost completely failed to elicit any calcium spikes (Figure 5F), although ACh 60, 200 nM still induced calcium increases, the calcium responses were markedly diminished in magnitude (Figure 5F). Analysis of the CCK- or ACh-stimulated calcium responses, in AR4-2J cells cultured alone or in AR4-2J cells co-cultured with pancreatic stellate cell, confirmed that the co-culture inhibition was statistically significant at all CCK (Figure 5G) or ACh (Figure 5H) concentrations used.

Pancreatic stellate cells have been estimated to account for 4–7% of parenchymal cells in the pancreas in vivo, the ratio of pancreatic acinar to stellate cell ratio has been calculated to be 13:1 to 24:1, although elongated cytoplasmic protrusions of pancreatic stellate cells could largely cover the basal plasma membrane surface of acinar cells in the acinus configuration [22,30]. Therefore in the following series of experiments, in co-culture the AR4-2J: pancreatic stellate cell ratio was set at 2:1, 10:1 or 50:1, which are likely to cover the full physiological range of acinar to stellate cell ratios.

### 3.5. Modulations of Stellate Cell Co-Culture Inhibition of AR4-2J Cell Calcium Signaling

**Figure 6:**
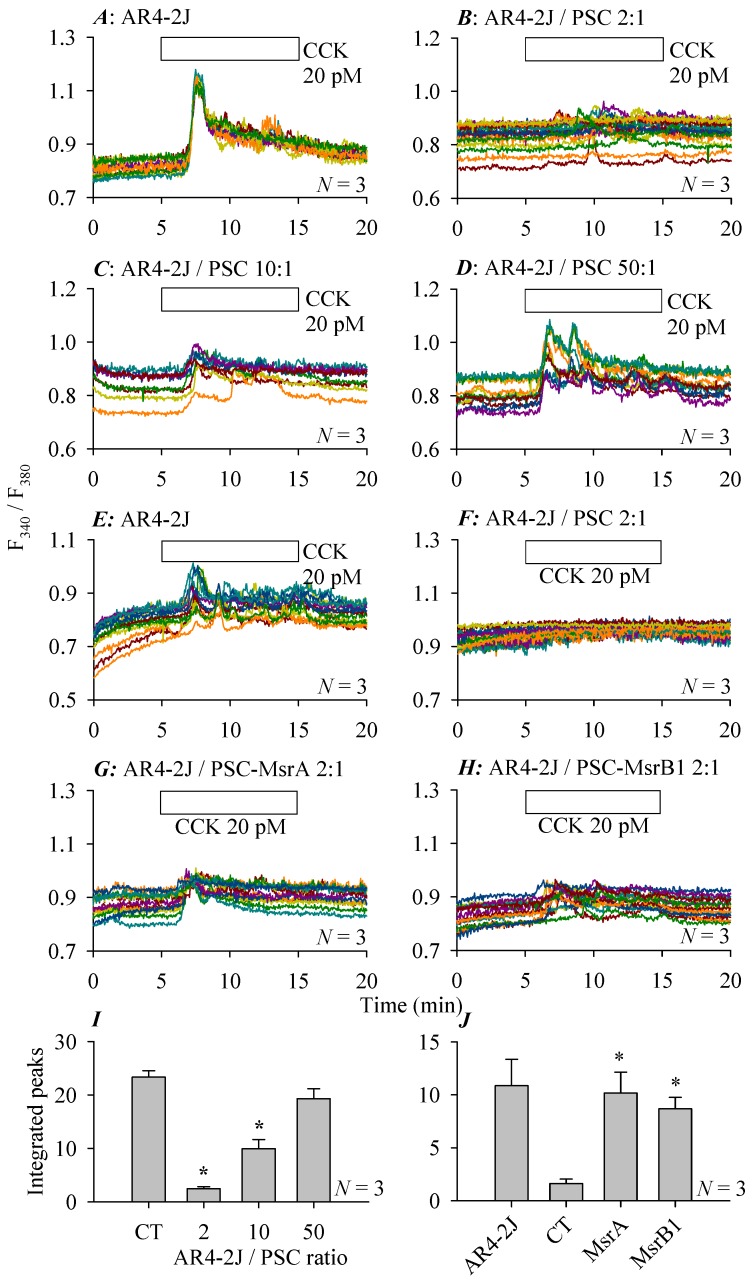
Co-culture inhibition of CCK-induced calcium oscillations in AR4-2J: Modulation by AR4-2J/PSC ratio or stellate cell Msr over-expression. AR4-2J cells cultured alone (**A**,**E**) or AR4-2J cells co-cultured at AR4-2J:PSC ratios of 2:1 (**B**,**F**–**H**), 10:1 (**C**), 50:1 (**D**) at 48 h (**A**–**D**) or 24 h (**E**–**H**) of AR4-2J seeding or co-culture, cells were loaded with Fura-2 AM. Pancreatic stellate cells used for co-culture with AR4-2J in (**F**–**H**) were transfected with either blank vectors (**F**) or with vectors containing gene for MsrA (**G**) or MsrB1 (**H**). Fura-2-loaded cells were perfused, CCK 20 pM (5–15 min) was added as indicated by the horizontal bars. Color-coded calcium tracings (**A**–**H**) in each panel are from individual AR4-2J cells from representative experiments with AR4-2J cultured alone or with AR4-2J/PSC co-culture. Experiments in (**A**–**D**) and (**E**–**H**) were done in time-matched parallel respectively. Quantification of CCK-induced calcium oscillations (integrated calcium peaks above baselines, min 5–15) from (**A**–**D**) or from (**E**–**H**) were plotted in (**I**) or (**J**) respectively. Asterisks (*) indicate statistical significance between AR4-2J alone (**A**, CT, control) and AR4-2J/PSC co-culture at different ratios (2, 10, 50, **B**–**D**) in (**I**) or between AR4-2J cells co-cultured with pancreatic stellate cells transfected with blank vectors (**F**, CT, control) and AR4-2J cells co-cultured with pancreatic stellate cells over-expressing MsrA (**G**) or MsrB1 (**H**) in (**J**), at *p* < 0.05, *N* = 3.

As shown above, CCK 20 pM could induce a strong calcium response in AR4-2J cells cultured alone (Figure 6A). With an AR4-2J: PSC co-culture ratio of 2:1, CCK failed nearly completely to induce calcium increases in AR4-2J cells (Figure 6B). With an AR4-2J: PSC ratio of 10:1, CCK-induced calcium oscillations in AR4-2J cells were still much diminished (Figure 6C). With an increased AR4-2J:PSC ratio of 50:1, inhibitory co-culture effect was no longer seen, CCK induced sizable calcium oscillations in co-cultured AR4-2J cells (Figure 6D), similar in size to the response seen in AR4-2J cells cultured alone (Figure 6A). Therefore compared with pure AR4-2J cells, AR4-2J/PSC co-culture at the ratios of 2, 10 resulted in statistically significant inhibitions of CCK-induced calcium response but at the ratio of 50 the inhibition was no longer significant (Figure 6I). These correlations between acinar/stellate cell ratio and the extent or intensity of inhibition are probably of physiological significance. With the normal or physiological pancreatic acinar/stellate cell ratio of 13:1 to 24:1 in vivo [30], there would be plenty space for the stellate cells to exert significant and varied levels of inhibition on CCK- or ACh-stimulated calcium responses in normal pancreatic acinar cells.

It is unclear how pancreatic acinar/stellate cell co-culture could have led to inhibited calcium responses in pancreatic acinar cell AR4-2J. Most remarkably, in an additional series of experiments, it was found that Msr over-expression in the co-cultured pancreatic stellate cells could overcome this co-culture inhibition. In this series of experiments, time-matched experiments revealed that in AR4-2J cells cultured alone, CCK 20 pM induced normal calcium oscillations (Figure 6E). AR4-2J co-culture (at AR4-2J/PSC ratio of 2:1) resulted in a complete blockade of CCK-induced calcium oscillations in AR4-2J cells (Figure 6F). However, if pancreatic stellate cells used in co-culture over-expressed MsrA (Figure 6G) or MsrB1 (Figure 6H), CCK-induced calcium responses in the co-cultured AR4-2J cells were conspicuously restored. Analysis of the CCK-induced calcium responses (integrated calcium peaks above basal levels from 5–15 min) in (Figure 6E–H) revealed that in comparison with co-culture with pancreatic stellate cells transfected with blank vectors only, co-culture with pancreatic stellate cells over-expressing MsrA or MsrB1 led to statistically significant increases in CCK stimulated calcium responses in AR4-2J cells (Figure 6J). For these experiments, pancreatic stellate cells were transfected either with blank adenovirus vector (Figure 6F) or with adenovirus vectors containing genes for MsrA (Figure 6G) or MsrB1 (Figure 6H). The transfected pancreatic stellate cells were co-cultured with AR4-2J cells for 24 h before calcium measurements were performed.

**Figure 7:**
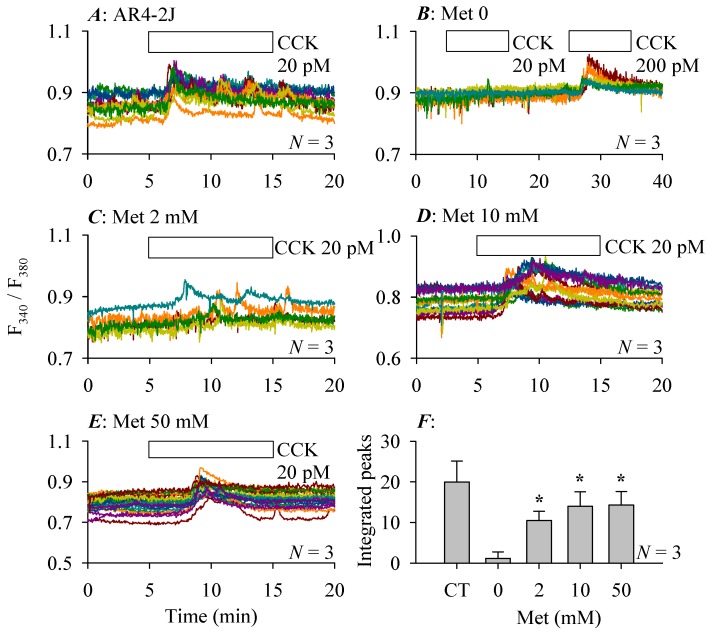
Co-culture inhibition of CCK-induced calcium oscillations in AR4-2J cells: reversal by Met supplementation in the co-culture medium. AR4-2J cells cultured alone (**A**) or AR4-2J co-cultured with PSC (**B**–**E**) at 48 h of co-culture (AR4-2J/PSC 2:1) were loaded with Fura-2 and perfused, CCK 20 pM was added as indicated by the horizontal bars. The co-culture medium was supplemented with Met at 0 (**B**), 2 (**C**), 10 (**D**), 50 mM (**E**) at 24 h before calcium measurements. The color-coded calcium tracings in each panel (**A**–**E**) are from individual AR4-2J cells from a typical experiment. CCK-stimulated calcium oscillations were analyzed (integrated calcium peaks above baselines from min 5–15) and plotted in (**F**), with comparisons made between co-cultured AR4-2J with no Met (**B**) and Met at 2 (**C**), 10 (**D**), 50 mM (**E**) respectively, asterisks (*) indicate statistical significance at *p* < 0.05, *N* = 3.

Other than Msr over-expression, it was found that co-culture inhibition of CCK-stimulated calcium responses in AR4-2J cells could also be rescued by Met supplementation in the co-culture medium. In this series of experiments, time-matched control experiments revealed that in AR4-2J cells cultured alone, CCK 20 pM induced regular calcium increases (Figure 7A). Co-culture with pancreatic stellate cells led to complete inhibition of CCK 20 pM-triggered calcium increases in AR4-2J cells; but in these same cells a subsequent higher CCK concentration of 200 pM did induce significant calcium increases, indicating a much reduced CCK1 receptor sensitivity towards CCK (Figure 7B). The pancreatic stellate cell co-culture-suppressed AR4-2J cell calcium oscillations were partially restored by supplementation in the co-culture medium of Met 2 mM for 24 h (Figure 7C). Higher Met concentrations of 10 mM (Figure 7D) or Met 50 mM (Figure 7E) for 24 h in the co-culture medium resulted in more complete restorations of CCK-induced calcium responses in AR4-2J cells. Compared with the near complete abolishment of CCK-induced calcium increases without Met, Met supplementation restored CCK-induced calcium responses were statistically significant at all Met concentrations used (Figure 7F).

## 4. Discussion

We have found in the present work that MsrA, B1 and B2 were expressed in the freshly isolated rat pancreatic stellate cells, Msr expression diminished after activation in culture. Msr over-expression enhanced ATP-induced calcium increases but inhibited reactive oxygen species generation and IL-1 synthesis/release in pancreatic stellate cells. Rat pancreatic stellate cell co-culture with pancreatic acinar tumor cell AR4-2J inhibited CCK- and ACh-stimulated calcium increases in AR4-2J cells. Co-culture inhibition of CCK-stimulated calcium increases in AR4-2J cells was modulated by AR4-2J/PSC ratio (2, 10, to 50). Further, co-culture inhibition of CCK stimulated calcium responses in AR4-2J cells were restored by over-expression of MsrA, B1 in the pancreatic stellate cells and by Met (2, 10, 50 mM) supplementation in the co-culture medium. These data together suggest that Msr play a major role not only in pancreatic stellate cell physiology but also in stellate/acinar cell interactions. The strategically located pancreatic stellate cells (located at the basal plasma membrane of the acinus where acinar cell surface receptors are expressed) may therefore provide a brake mechanism on pancreatic acinar cell signaling such as calcium oscillations, the brake mechanism is further modulated both by acinar/stellate cell ratio and by the level of stellate cell Msr expression.

The isolated rat pancreatic stellate cells were found to express MsrA, B1, B2 (Figure 1) but not MsrB3 (not shown). MsrA expression has been reported in other tissues such as brain, liver, testes, heart and lungs [46]. MsrB1 is known to be expressed in liver and kidney [9]. MsrB2 is expressed in heart, kidney, retina and skeletal muscle [47]. MsrB3 is expressed mainly in the auditory and vestibular sensory epithelial cells in the inner ear [48].

MsrA, B1, B2 expression all declined, in a bi-phasic fashion, in pancreatic stellate cells in culture in comparison with the freshly isolated pancreatic stellate cells (Figure 1). The first phase of decline in MsrA, B1, B2 expression all occurred on culture day 1 but the second longer-lasting phase of decline started on day 14 for MsrA but on day 7 for MsrB1, B2. MsrB2 expression decreased more (almost down to 50% of the initial value) than MsrA or MsrB1 (Figure 1B–D). It may be noted here that the second phase of decline in Msr expression occurred after the expression of α-smooth muscle actin was activated (i.e., culture activation of pancreatic stellate cells, which occurred on day 5, data not shown). Although we are not completely certain about the reason for this bi-phasic decline, we were keen to maintain a high level of Msr expression to examine their possible functions in pancreatic stellate cells. Packaged adenovirus vectors were used for this purpose. Data indicate that the packaged adenovirus vectors were all effective to transfer the Msr genes to primary cultured rat pancreatic stellate cells (Figure 2). Compared to retroviral vectors, recombinant adenovirus vectors do not integrate into the host genome [49] and has been used for both short- and longer-term gene transfers into primary cells [50] or even as potential therapeutics against cancer, cardiovascular and infectious diseases [51]. The adenovirus vectors can be readily targeted to the pancreatic stellate cells in vitro. For example, previously the *IL-4* gene was transferred into pancreatic stellate cells with packaged adenovirus [52]. In the present work we found that adenovirus-mediated Msr over-expression had marked effect on pancreatic stellate cell physiology.

At 24 h after adenovirus infection of cultured rat pancreatic stellate cells, MsrA, B1 or B2 expression levels were all conspicuously elevated as confirmed by RT-PCR, Western blot and immunocytochemistry (Figure 2). Note that MsrB1 protein overexpression seemed to be less pronounced than MsrA and MsrB2 (Figure 2D). Since MsrB1 is a selenoprotein [53], this probably indicates that although baseline MsrB1 protein expression is normal, for optimal MsrB1 over-expression, additional Se supplementation might be required. Immunocytochemistry revealed that MsrA, B1, B2 were expressed in cytoplasm in both freshly isolated and cultured pancreatic stellate cells, consistent with previous reports about their subcellular localizations in other cell types: MsrA in mitochondria, cytosol and nucleus, MsrB1 in cytosol, nucleus, MsrB2 in mitochondria and MsrB3A in ER, mitochondria [53]. Note the puncta pattern of over-expressed MsrB1, B2 in particular, which might suggest mitochondrial localizations (Figure 2).

Rat pancreatic stellate cells typically respond to ATP stimulation by an increase in cytosolic calcium concentration, due to activation of purinergic receptors [54,55]. ATP-induced increases in cytosolic calcium in pancreatic stellate cells were markedly enhanced by the over-expression of MsrA, B1 or B2 (Figure 3A–E). MsrA over-expression has previously been found to increase complex IV activity of the mitochondrial respiratory chain, to produce more ATP molecules [56]. Over-expression of MsrB1 or B2 may have similar effect, together with over-expressed MsrA, to produce more ATP, to all stimulate enhanced calcium increases (Figure 3A–E). Since in the present work the pancreatic stellate cells were continuously perfused, enhanced calcium responses seen might also be due to direct sensitization of the purinergic receptor or related signaling proteins involved in calcium signaling.

In contrast to their sensitization of purinergic stimulation by ATP, over-expressions of MsrA, B1 or B2 were all found to decrease baseline reactive oxygen species generation in pancreatic stellate cells (Figure 3F–H), which could potentially be due to Msr modulation of NADPH oxidase activity. Therefore MsrA, B1, B2 expression or over-expression lend to a more reduced state in the pancreatic stellate cells. Others have reported previously that MsrA over-expression reduced LPS-stimulated reactive oxygen species production in microglia [18]. A more reduced state after Msr over-expression is likely to lead to less activation of pancreatic stellate cells because reactive oxygen species are known to be major activators of pancreatic stellate cells [57]. Indeed MsrA, B1, B2 over-expressions all resulted in significantly reduced content of α-smooth muscle actin in the cultured pancreatic stellate cells (data not shown).

As mentioned before pancreatic stellate cells all express the cell surface receptor TLR4 [42,43,44,45]. TLR4 agonist LPS was found to induce dose-dependent expression of IL-1, Jak and STAT3 in pancreatic stellate cells, especially of IL-1 (Figure 4A–C). Over-expression of MsrA, B1 or B2 was found to inhibit almost completely LPS-induced IL-1 expression and synthesis (Figure 4D,E). Msr over-expression may, by inhibiting pancreatic stellate cell synthesis and release of inflammatory IL-1, provide a mechanism to counter inflammation or development of chronic pancreatitis. TLR4 has been found to mediate activation of human pancreatic stellate cells [42]. TLR4 agonist LPS was found to contribute to the development of pancreatic fibrosis, silencing TLR4 expression in pancreatic stellate cells was found to alleviate pancreatic fibrosis [45]. Msr over-expression might therefore overcome these changes that are essential in the development of chronic pancreatitis.

Activated pancreatic stellate cells have also been found to be important for niche formation in pancreatic ductal adenocarcinoma [58], which was projected to be the second deadliest cancer by 2030 [59]. Msr expression-modulated activation of the pancreatic stellate cell function might provide a useful strategy in treating such cancers.

Other than modulating pancreatic stellate cell function, pancreatic stellate cell Msr over-expression was found to also exert a major effect on pancreatic acinar cell function in pancreatic acinar/stellate cell monolayer co-culture.

Both CCK and ACh readily induced calcium oscillations in rat pancreatic acinar cell AR4-2J cultured alone but such CCK- and ACh-induced calcium responses were markedly inhibited after co-culture with rat pancreatic stellate cells (Figure 5). Such stellate cell-mediated inhibition of receptor-mediated calcium oscillations in pancreatic acinar cells would probably lead to a blockade of zymogen granule exocytosis/secretion in the exocrine pancreas. This would probably provide the third alternative mechanism for blocked zymogen secretion associated with acute pancreatitis: (***a***) secretion blockade due to neutrophil respiratory burst [35], (***b***) secretion blockade due to extracellular histone inhibition of CCK or ACh stimulation [41] and (***c***) secretion blockade due to pancreatic stellate cells (current work). Therefore at least triple mechanisms exist to decelerate the activation of pancreatic acinar cell surface receptors, receptor-mediated calcium signaling and digestive enzyme secretion. Of these three independent mechanisms, blockade by neutrophils and by pancreatic stellate cells are both dependent on their cell density [35] (Figure 6A–D,I). Importantly, blockade by pancreatic stellate cells is also dependent on stellate cell Msr expression level (Figure 6E–H,J). In sum, at least three independent mechanisms in two cell types are in place to ensure that pancreatic acinar cells are not over-stimulated in vivo. Nature has provided all the safety checks that are necessary to prevent over-drive or over-stimulation of pancreatic acinar cells. Further, over-stimulation of pancreatic acinar cells leads to supra-maximal inhibition in digestive enzyme secretion [39,60,61]. Therefore pancreatic acinar cell secretion is subject to multiple levels of inhibitory regulation.

Rat pancreatic stellate cell co-cultured with AR4-2J cells suppressed CCK- or ACh-induced AR4-2J cell calcium oscillations (Figure 6A–D,I), as mentioned above. This inhibition was dependent on the relative ratio of AR4-2J/PSC, fewer stellate cells leading to progressively weaker inhibitory effect (Figure 6A–D,I). This dependence on relative pancreatic stellate cell density is similar to that of neutrophil inhibition of CCK- or ACh-induced calcium oscillations. Neutrophil respiratory burst from 5 × 10^5^/mL neutrophils (activated by fMLP 10 μM) was found to block calcium increases elicited by CCK 10 pM [35] but respiratory burst from a reduced density of neutrophils (1.67 × 10^5^/mL activated by fMLP 10 μM) had little inhibitory effect on CCK-induced calcium oscillations [35].

Pancreatic acinar/stellate cell interactions have also been reported before in a mouse pancreatic acinar/stellate cell co-culture system, where pancreatic acinar cells were planted in the culture wells and pancreatic stellate cells in inserts therefore acinar and stellate cells do not come into direct physical contact. Even without any physical contact between these two cell types, caerulein (100 pM)-induced amylase secretion in mouse pancreatic acinar cells was suppressed in the co-culture [62]. In the mouse co-culture system, whether the failed caerulein stimulation of amylase secretion in the primary cultured mouse pancreatic acinar cells was due to inhibited calcium signaling was not known. Interestingly, hepatic stellate cells/neutrophils co-culture was reported recently to prolong neutrophil survival [63]. But whether Msr was involved in those two earlier co-culture systems (mouse pancreatic acinar/stellate cells, hepatic stellate cells/neutrophils) was not determined.

The co-culture inhibition of CCK-induced calcium oscillations in AR4-2J cells was rescued or restored by Msr over-expression in the co-cultured pancreatic stellate cells. Over-expression of either MsrA or B1 was effective to restore CCK-induced calcium oscillations (Figure 6E–H,J). This further underlines the important role of stellate cell Msr expression in calcium signaling and possibly also digestive enzyme secretion [62] in the pancreatic stellate cell-surrounded pancreatic acinus. Although the detailed molecular mechanism or chemical basis of the rescue of pancreatic acinar cell calcium signaling by pancreatic stellate cell Msr overexpression is not certain at this point, it is noteworthy that pancreatic stellate cell MsrA, B1, B2 overexpression all diminished both production of reactive oxygen species (Figure 3F–H) and secretion of IL-1 (Figure 4E) by the Msr-overexpressing pancreatic stellate cells. These diffusible molecules, among other factors secreted from the stellate cells, are likely to play important roles in the observed co-culture inhibition.

To further confirm a role of pancreatic stellate cell Msr expression in the modulation of pancreatic acinar cell function, addition of free Met (2–50 mM, added 24 h before CCK stimulation) to the AR4-2J/PSC co-culture medium was found to readily rescue co-culture inhibition of CCK-induced calcium oscillations in AR4-2J (Figure 7). Enhanced free Met concentrations would take pressure off the Msr so that more Met(O) would be converted back into Met in the protein peptide chains. Therefore these data further corroborate a role of pancreatic stellate cell Msr in the modulation of acinar cell function.

Relevant to the role of pancreatic stellate cell Msr expression was a couple of recent reports on the effect of supplementation of Met-containing antioxidants in diet in a cohort of chronic pancreatitis patients. Met supplementation was found to alleviate chronic pancreatic pain in most of the patients studied in these works [64,65].

Relevant to the co-culture inhibition of rat pancreatic acinar cell calcium signaling by rat pancreatic stellate cells (Figure 5) and their rescue by rat pancreatic stellate cell Msr over-expression (Figure 6) and by Met supplementation in the co-culture medium (Figure 7) and relevant to co-culture inhibition of amylase secretion in mouse pancreatic acini by mouse pancreatic stellate cells reported previously [62], it may be noted here that amylase secretion in rat pancreatic acini has been reported to be stimulated by human pancreatic stellate cells, up to 20% of basal secretion (from basal of 100% to stimulated of 120%), after rat pancreatic acini/human pancreatic stellate cell co-incubation (at an acinar: stellate cell ratio of 6.7:1) for 30 min [66]. Since basal amylase secretion is typically at about 5% of total pancreatic acinar cell amylase content in a good preparation of intact pancreatic acini [60,61,62], this would amount to a stimulation from 5% of total amylase content in control pancreatic acini to 6% of total amylase content in pancreatic stellate cell-stimulated pancreatic acini, which is well within the standard error range of 2% of total amylase content normally found in amylase assay [60]. Such a miniscule stimulatory effect is rather negligible in comparison to the stimulation by CCK or ACh which is typically at about 25% or more of total amylase content [60,61]. Such slight stimulation might be obscured, obliterated or made completely irrelevant by the pancreatic acinar/stellate cell co-cultured inhibition of CCK or ACh stimulation of pancreatic acinar cells (note that co-culture inhibition was significant at acinar: stellate cell ratios of 2:1 and 10:1, see Figure 6. The ratio of 6.7:1 is within this ratio range). In nature, *rat* pancreatic acinar cells probably never come into contact with *human* pancreatic stellate cells, not even for 30 min. Also longer term co-culture (for several days) of pancreatic acinar cells with pancreatic stellate cells from the same species may be needed to reflect more faithfully the interaction between rodent pancreatic acinar cells and pancreatic stellate cells in vivo. Nevertheless, the present work would not be possible without previous publications on pancreatic stellate cells which laid the foundation for more detailed investigations.

## 5. Conclusions

**Figure 8:**
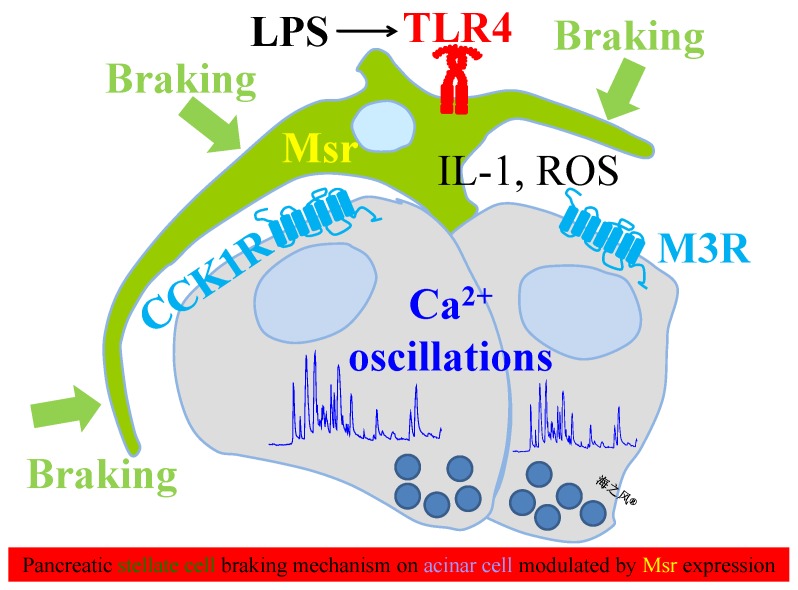
Rat pancreatic stellate cells serve as a brake mechanism on pancreatic acinar cell calcium signaling modulated by Msr expression. Activation of pancreatic acinar cell cholecystokinin 1 (CCK1) or muscarinic 3 (M3) acetylcholine receptors triggers cytosolic calcium oscillations, which can be blocked by the surrounding activated pancreatic stellate cells. LPS stimulation of rat pancreatic stellate cell surface TLR4 triggers reactive oxygen species generation and IL-1 production, leading to the activated state of pancreatic stellate cells. Pancreatic stellate cell activation is inhibited by the over-expression of Msr to decelerate the braking mechanism. Note that both IL-1 and reactive oxygen species (ROS) could readily diffuse from stellate to acinar cells.

In conclusion, rat pancreatic stellate cells express MsrA, B1, B2, which diminishes after stellate cell activation in culture. Over-expression of MsrA, B1 or B2 enhances ATP-stimulated calcium increases but reduces reactive oxygen species generation and inhibits LPS-induced/TLR4-mediated secretion of pro-inflammatory IL-1. Rat pancreatic stellate cell co-cultured with AR4-2J inhibits CCK- or ACh-elicited calcium oscillations in AR4-2J dependent on acinar/stellate cell ratio but are restored by over-expression of MsrA, B1 or B2 in pancreatic stellate cells and by supplementation of Met in the co-culture culture medium. These data together suggest that Msr play important roles in rat pancreatic stellate cell physiology. The pancreatic stellate cells due to their strategic location near the basal plasma membrane of the surrounded acinar cells may serve as a brake mechanism on pancreatic acinar cell calcium signaling with modulation by stellate cell Msr expression (Figure 8). All the G protein-coupled receptors are located on the basal plasma membrane in pancreatic acinar cells. The basal plasma membrane is also the place where neurotransmitters and hormones exert their regulatory roles on receptor-mediated secretion of the pancreatic acinar cells. Therefore it is only logical that the pancreatic stellate cells should exert a brake mechanism at or near the basal plasma membrane of the pancreatic acinar cells.

## Figures and Tables

**Figure A1:**
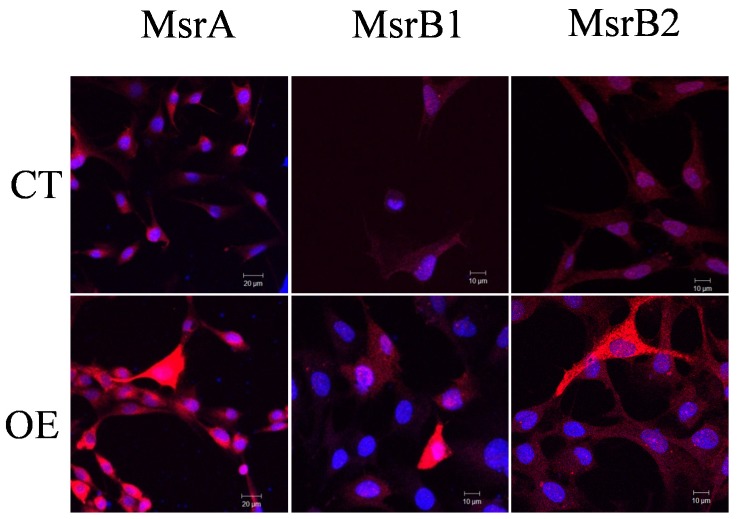
Msr over-expression in cultured rat pancreatic stellate cells. Pancreatic stellate cells were transfected with adenovirus vectors and immunocytochemistry was performed as described in Figure 2E. The primary anti-MsrA, B1, 2 antibodies and TRITC labeled donkey anti-rabbit secondary antibody were used. Nucleus was counter-stained with Hoechst 33342. Imaging was done in a Zeiss LSM 510 META (objective 63×/1.40 oil) with λ_ex_: TRITC/543 nm, Hoechst 33342/405 nm. No fluorescence was observed in time-matched control experiments (without primary and secondary antibodies, without primary antibody but with secondary antibody, non-specific rabbit IgG as primary antibody plus secondary antibody, not shown). The laser power for fluorescent excitation was set as in Figure 1E,F but not tuned down as in Figure 2E even for the Msr-overexpressing (OE) pancreatic stellate cells. Note the over-exposed staining pattern in Msr overexpressing (OE) rat pancreatic stellate cells, Msr staining was normal in control pancreatic stellate cells (CT) not overexpressing Msr. The scale bars are 20 for MsrA, 10 μm for MsrB1, B2 respectively.

**Figure A2:**
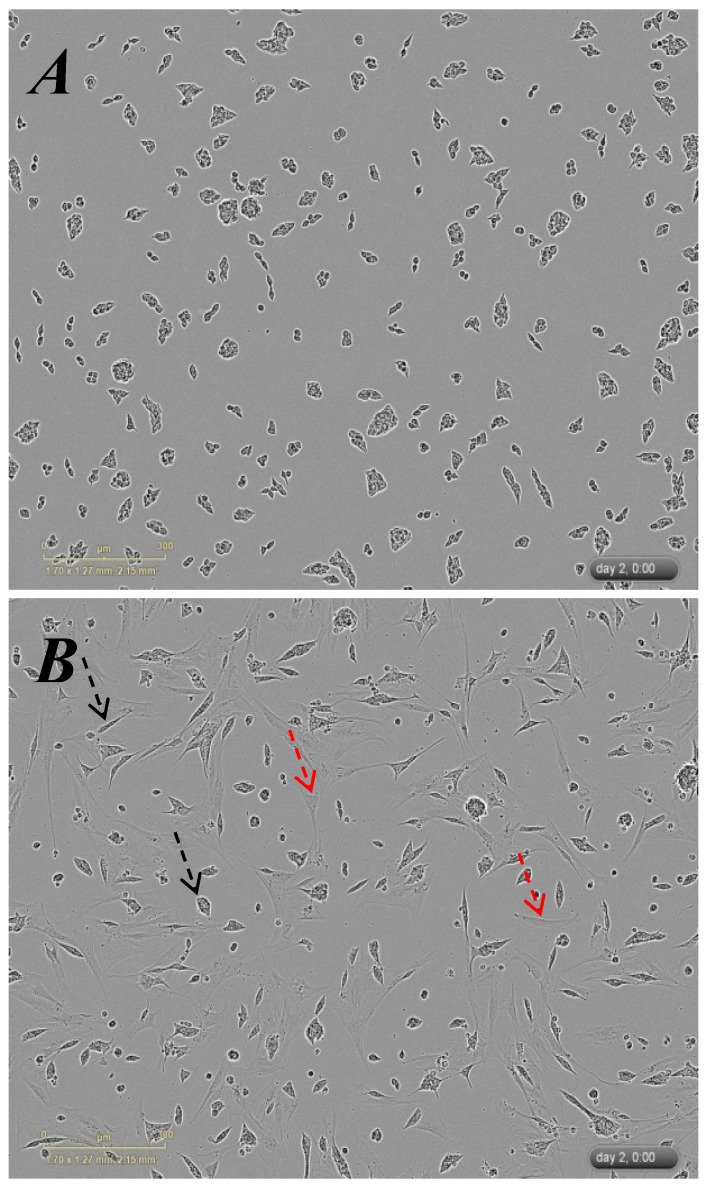
AR4-2J/PSC co-culture. AR4-2J cells cultured alone (**A**) or co-cultured with pancreatic stellate cells (at AR4-2J/PSC ratio of 2:1) (**B**) at 48 h of culture or co-culture (imaged by Incucyte). Black and red arrows indicate AR4-2J and pancreatic stellate cells respectively in the co-culture (**B**). These micrographs are enlarged versions from Figure 5A,B. The scale bars are 300 μm in (**A**,**B**).
